# Inhibition of poly(ADP-ribose) polymerase-1 or poly(ADP-ribose) glycohydrolase individually, but not in combination, leads to improved chemotherapeutic efficacy in HeLa cells

**DOI:** 10.3892/ijo.2012.1740

**Published:** 2012-12-17

**Authors:** XIAOXING FENG, DAVID W. KOH

**Affiliations:** Department of Pharmaceutical Sciences, College of Pharmacy, Washington State University, Pullman, WA 99164-6534, USA

**Keywords:** poly(ADP-ribose) glycohydrolase, poly(ADP-ribose) polymerase inhibitor, poly(ADP-ribose), chemotherapy, cell death

## Abstract

The genome-protecting role of poly(ADP-ribose) (PAR) has identified PAR polymerase-1 (PARP-1) and PAR glycohydrolase (PARG), two enzymes responsible for the synthesis and hydrolysis of PAR, as chemotherapeutic targets. Each has been previously individually evaluated in chemotherapy, but the effects of combination PARP-1 and PARG inhibition in cancer cells are not known. Here we determined the effects of the inhibition of PARP-1 and the absence or RNAi knockdown of PARG on PAR synthesis, cell death after chemotherapy and long-term viability. Using three experimental/clinical PARP-1 inhibitors in PARG-null cells, we show decreased levels of PAR and increased short-term and long-term viability with each inhibitor, with the exception of DPQ. Treatment with the experimental chemotherapeutic agent, N-methyl-N’-nitro-N-nitrosoguanidine (MNNG), led to increased cell death in PARG-null cells, but decreased cell death when pretreated with each PARP-1 inhibitor. Similar results were observed in MNNG-treated HeLa cells, where RNAi knockdown of PARG or pretreatment with ABT-888 led to increased HeLa cell death, whereas combination PARG RNAi knockdown + ABT-888 failed to produce increased cell death. The results demonstrate the ability of the PARP-1 inhibitors to decrease PAR levels, maintain viability and decrease PAR-mediated cell death after chemotherapeutic treatment in the absence of PARG. Further, the results demonstrate that the combination of PARP-1 and PARG inhibition in chemotherapy does not produce increased HeLa cell death. Thus, the results indicate that inhibiting both PARP-1 and PARG, which both are chemotherapeutic targets that increase cancer cell death, does not lead to synergistic cell death in HeLa cells. Therefore, strategies that target PAR metabolism for the improved treatment of cancer may be required to target PARP-1 and PARG individually in order to optimize cancer cell death.

## Introduction

The chemotherapeutic potential for targeting the metabolism of poly(ADP-ribose) (PAR) biopolymers in cancer cells is recognized due to the fundamental role PAR has in maintaining genomic integrity (1–3). After DNA-damaging chemotherapeutic agent treatments, PAR is synthesized primarily by PAR polymerase-1 (PARP-1) and PARP-2 (4,5). While the basal levels of PAR in untreated cells is low, the catalytic activity of PARP-1 can increase PAR levels up to 200-fold (6). Once synthesized, PAR is catabolized by the high specific activity of PAR glycohydrolase (PARG), the primary enzyme that catalyzes hydrolysis of PAR (7,8). This metabolism of PAR is important for the facilitation of efficient DNA repair (9,10) or the promotion of cell death (11,12), two essential outcomes necessary for the maintenance of genomic integrity after DNA damage. Thus, targeting PARP-1 and PARG are strategies to inhibit their genome-protecting effects in cancer cells and thereby induce cancer cell death. Further, targeting the metabolism of PAR was shown to preferentially lead to deleterious effects in rapidly dividing cells (13), which indicates an ability to specifically target cancer cells.

PARP-1 is an established chemotherapeutic target. This was based initially on the observation that inhibition of PARP-1 successfully inhibits DNA base-excision repair, which thus leads to increased DNA strand breaks after chemotherapeutic treatments (14,15). Subsequently, several generations of PARP inhibitors were developed and evaluated as radio- or chemosensitizers for the improved chemotherapeutic treatment of various types of cancers (16–18) (reviewed in ref. 19). Further, later studies demonstrated the ability of PARP inhibitors to successfully target tumors that exhibit mutations in the breast cancer 1, early onset protein (BRCA1), a tumor suppressor protein that is involved in the maintenance of genomic integrity (20) and the breast cancer 2, early onset protein (BRCA2), a protein involved in DNA double strand break repair and homologous recombination (21). As a result, PARP-1 inhibitors are currently being evaluated in clinical trials for their ability to enhance the efficacy of chemotherapeutic agents in ovarian and breast cancer patients that have BRCA-deficient tumors (22–26).

PAR glycohydrolase, on the other hand, is an emerging chemotherapeutic target. The absence of PAR hydrolysis due to the PARG-null mutation leads to remarkable phenotypes. *In vivo*, increased levels of PAR are observed, which leads to embryonic lethality (27–29). In cultured cells, the PARG-null mutation causes increased levels of DNA damage, enhanced levels of cell death, genomic instability and chemosensitization to sublethal doses of DNA-damaging agents (27,29,30). Similar effects utilizing PARG inhibitors or the RNAi knockdown of PARG were observed, where PARG silencing or inhibition led to radiation-induced mitotic catastrophe (31), chemosensitization in malignant melanoma cells (32) and targeted cell death in BRCA2-deficient tumor cells (33). In addition, although many PARPs have been identified and characterized for their ability to synthesize PAR in different cellular compartments and for their distinct roles in maintaining genomic integrity (5,34,35), PARG remains as the primary enzyme that catalyzes PAR hydrolysis. Accordingly, the targeting of PARG may modulate the genome-protecting roles of these other PARPs and thus potentially provide desired chemotherapeutic outcomes. Therefore, the targeting of PARG appears to be a feasible strategy to improve the chemotherapeutic treatment of cancer patients in the future.

However, it is not known if the targeting of both PARP-1 and PARG in cancer cells can lead to a synergistic increase in cancer cell death. While both enzymes have previously characterized roles in DNA repair, cell death and global genomic stability, studies evaluating the effects of combination therapy that utilizes the inhibition of both PARP-1 and PARG in chemotherapy are lacking. Here, we utilized the PARP-1-specific inhibitors 3,4-dihydro-5[4-(1-piperindinyl) butoxy]-1(2H)-isoquinoline (DPQ) (36), N-(6-oxo-5,6-dihydrophenanthridin-2-yl)-(N,N-dimethylamino)acetamide (PJ34) (37) and 2-((R)-2-methylpyrrolidin-2-yl)-1H-benzimidazole-4-carboxamide (ABT-888; Veliparib) (22), and the molecular genetic deletion or RNAi knockdown of PARG to determine the effects of combination PARP-1 and PARG inhibition. Our results demonstrate that the knockdown/inhibition of each PAR metabolic enzyme alone enhances chemotherapeutic effectiveness, but combination therapy does not lead to synergistic levels of cell death in HeLa cells. Thus, this study provides important insight into optimizing the chemotherapeutic strategies designed to improve the treatment of cancer patients via the targeting of PARP-1 and PARG.

## Materials and methods

### Materials

ABT-888 and DPQ were purchased from Enzo Life Sciences (Farmingdale, NJ). PJ34 was purchased from VWR (Radnor, PA). Benzamide was purchased from Sigma-Aldrich (St. Louis, MO). MNNG was from AccuStandard (New Haven, CT). RPMI-1640 media and fetal bovine serum (FBS) were purchased from Hyclone (Logan, UT). DMEM, RPMI and penicillin/streptomycin were purchased from Lonza (Walkersville, MD). Primary antibodies utilized were polyclonal anti-PAR (clone 96-10; Trevigen, Gaithersburg, MD), polyclonal anti-PARG (Millipore, Billerica, MA) and polyclonal anti-β-actin (Sigma-Aldrich). HRP-conjugated goat anti-rabbit IgG secondary antibody was purchased from Jackson ImmunoResearch (West Grove, PA). Annexin V-FITC was from SouthernBiotech (Birmingham, AL). Propidium iodide was purchased from Thermo Scientific (Pittsburgh, PA). Complete Mini, EDTA-free protease cocktail tablets were purchased from Roche (Indianapolis, IN).

### Cell culture

HeLa cells were obtained from American Type Culture Collection (ATCC; Manassas, VA) and cultured in DMEM + 10% FBS. Wild-type and PARG-null embryonic trophoblast stem (TS) cells were derived from E3.5 mouse blastocysts as previously described (29). PARG-null TS cells were maintained in TS growth medium containing RPMI, fibroblast growth factor-4 (R&D Systems, Minneapolis, MN), heparin sodium (Sigma-Aldrich), 50% murine embryonic feeder (MEF)-conditioned medium and 15% FBS. For long-term viability, PARG-null TS cells were maintained in TS growth medium containing 0.5 mM benzamide (BZ), a non-specific PARP inhibitor.

### Immunoblot analyses

PARG-null TS cells were grown in medium containing the PARP-1 inhibitors DPQ, PJ34 or ABT-888 [all dissolved in DMSO and kept as stock solutions of 10 mM (ABT-888) or 30 mM in small aliquots at −20°C] at the doses indicated. After 2 days, cells were harvested by trypsinization. Cells were then lysed by brief sonication in ice-cold lysis buffer (25 mM Tris-HCl pH 7.5, 150 mM NaCl, 1% NP-40, 1 mM EDTA, 1 mM EGTA and 1 protease inhibitor cocktail tablet) and the extracts were placed on ice for 30 min. After centrifugation (13,000 rpm for 15 min), proteins were denatured with SDS loading buffer at 100°C for 2 min. Samples (20 *μ*g) were subjected to 7.5% SDS-PAGE and transferred to nitrocellulose. Membranes were blocked with PBS + 0.1% Tween-20 containing 5% milk for 1 h and incubated with anti-PAR primary antibody (1:1,000) overnight at 4°C. Membranes were then washed with PBS-Tween three times and incubated with HRP-conjugated goat anti-rabbit antibody (1:10,000) for 1 h in PBS-Tween + 5% milk. The blot was washed as before and analyzed by Chemidoc XR gel imager (Bio-Rad, Hercules, CA). To verify equivalent protein levels, the membranes were stripped with Restore Western Blot Stripping buffer (Pierce, Rockford, IL) according to the manufacturer’s protocol and washed with PBS-Tween three times. The membranes were then re-blocked for 1 h, incubated with anti-β-actin primary antibody for 1 h at room temperature, washed and analyzed by gel imager.

To determine the short-term effects of the inhibition of PARP-1 catalytic activity and the absence of PARG, wild-type and PARG-null TS cells were grown in medium containing 20 *μ*M DPQ, 4 *μ*M PJ34 or 10 *μ*M ABT-888 for 1–5 days and immunoblot analyses of PAR levels were performed as above. For long-term effects, TS cells were passaged five times in medium containing the PARP-1 inhibitors for a total treatment period of 15 days. At the end of each passage, cells were harvested and lysed as before, and immunoblot analyses of PAR levels were performed. For both short- and long-term experiments, cells were provided fresh medium containing PARP-1 inhibitor every 2 days. After passage 1 and 2, cells were observed by a Nikon Diaphot microscope using the 10X objective.

### Cell death assay

PARG-null TS cells were grown in growth medium containing PARP-1 inhibitors for 2 days and treated with 50 *μ*M MNNG for 10 min. Cells were then washed once each with PBS and TS growth media and incubated in TS growth media containing the PARP inhibitors for 20–24 h. Cells were then harvested by trypsinization, washed once with ice-cold PBS, centrifuged briefly (1,000 rpm, 5 min, 4°C), and the pellet was resuspended with 0.1 ml 1X Annexin-binding buffer (VWR). To each sample, 2.5 *μ*l of Annexin V-FITC and 1 *μ*l of 100 *μ*g/ml propidium iodide was added. Samples were then incubated at room temperature and protected from light for 15 min. The samples were then analyzed using a BD AccuriC6 flow cytometer with blue/red lasers and four fluorescent detectors (Accuri, San Jose, CA). FL1 and FL3 channels were utilized to detect FITC and PI fluorescence. Cell death was determined by quantifying the total number of cells in the upper left, upper right and lower right quadrants of the FACS dot plots.

For treatment of HeLa cells, cells were seeded at 6×10^3^ cells/well and the next day, at approximately 70–80% confluence, cells were pretreated with 10 *μ*M ABT-888 for 30 min and/or RNAi knockdown of PARG 48 h prior to treatments. Cells were then treated with 0.5 mM MNNG for 30 min, the cells were washed with PBS, then washed with fresh growth medium. Cells were then cultured in growth medium containing ABT-888. After 24 h, cell death was analyzed by FACS.

### RNA interference

For the silencing of PARG by RNAi, HeLa cells were plated at 3×10^3^ cells/well in 24-well plates in antibiotic-free growth medium (DMEM + 10% FBS). The next day, at approximately 40% confluence, the medium was replaced with 0.3 ml Opti-MEM (Invitrogen, Carlsbad, CA). Cells were then transfected with 200 nM of small-interfering RNA (siRNA) duplex that targets bases 1832–1852 of human PARG mRNA (sequence: 5′-AAGATGAGAATGGTGAGCGAA-3′) (Integrated DNA Technologies, Ames, IA) using Oligofectamine (Invitrogen), according to the manufacturer’s standard protocol. Briefly, in separate mixtures, PARG siRNA was diluted in 50 *μ*l Opti-MEM and 6 *μ*l Oligofectamine was diluted in 24 *μ*l Opti-MEM. After 20 min, the mixtures were combined, gently mixed and added (dropwise) into the wells. A scrambled version of siRNA (5′-AGACAGAAGACAGAUAGGC-3′) was used as negative control. After 24 h, the medium was replaced with normal growth medium. PARG levels were analyzed by immunoblot 48 h after transfection using polyclonal anti-PARG primary antibody at a dilution of 1:1,000.

### Statistics

All error bars represent the standard error of the mean (SEM). Statistical analyses performed included one-way analysis-of-variance (ANOVA) and unpaired Student’s t-test. P-values <0.05 were considered statistically significant. All FACS samples were run in triplicate and experiments were performed at least three times.

## Results

### Decreased levels of PAR in PARG-null cells treated with PARP-1 inhibitors

**Figure 1.**
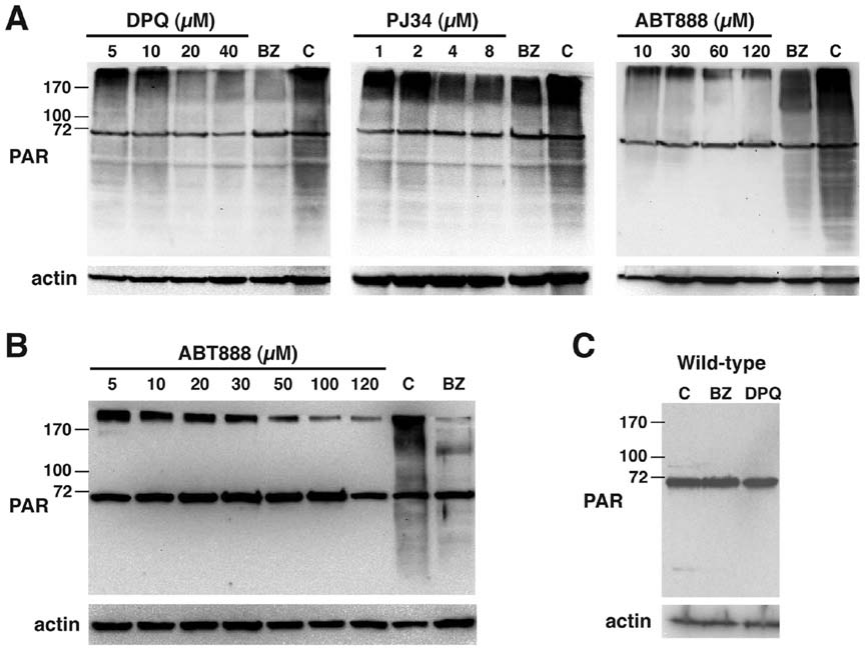
Poly(ADP-ribose) (PAR) levels in PARG-null cells treated with PARP-1 inhibitors. (A) PARG-null cells were cultured with increasing doses of the PARP-1 inhibitors DPQ, PJ34 and ABT-888. After 2 days, cells were harvested and PAR levels were analyzed by immunoblot using polyclonal anti-PAR (clone 96-10). Positive control for PARP inhibition was provided by PARG-null cells cultures in 0.5 mM benzamide (BZ), a non-specific first generation PARP inhibitor. Negative control (C) was provided by untreated PARG-null cells. Verification of equivalent protein levels was provided by the immunoblot detection of β-actin. (B) The ability of ABT-888 was further evaluated for its ability to inhibit PAR levels in PARG-null cells by utilizing a dose range of 5–120 *μ*M. PAR levels were analyzed by immunoblot and controls were equivalent as in (A) above. (C) Wild-type cells were treated with 10 *μ*M DPQ and PAR levels were analyzed by immunoblot. Note: the protein band near 66 kDa in all lanes of each blot represents the immunodetection of bovine serum albumin (BSA), which was utilized as a hapten in the production of the anti-PAR antibody.

To characterize the ability of PARP-1 inhibitors to inhibit PAR levels, we analyzed the ability of three PARP-1 inhibitors to reduce levels of PAR in PARG-null cells. It is worth noting that these PARP-1 inhibitors also inhibit PARP-2, but PARP-1 is known to catalyze the synthesis of the vast majority of PAR in the cell (38). We previously demonstrated that, in the absence of cell stress, PAR levels gradually accumulate in PARG-null cells (29). As a result, PARG-null cells are required to be grown in the presence of benzamide (BZ), a first generation non-specific PARP inhibitor, for long-term viability. Here, we cultured PARG-null cells in the presence of specific PARP-1 inhibitors. The results demonstrate that increasing doses of DPQ (5–40 *μ*M), PJ34 (1–8 *μ*M) and ABT-888 (10–120 *μ*M) led to dose-dependent decreases in the levels of PAR in PARG-null cells (Fig. 1A). DPQ and PJ34 both decreased PAR amounts to a similar level observed after BZ treatment. However, ABT-888 produced the greatest level of PAR reduction. Further analysis demonstrated a clear ability of ABT-888 to decrease PAR levels in a dose-dependent (5–120 *μ*M) manner (Fig. 1B). No accumulation of PAR was observed in wild-type cells in the absence or presence of BZ or DPQ (Fig. 1C), which shows the ability of these cells to catalyze the hydrolysis of PAR, as they contain functional PARG. These results demonstrate the ability of PARP-1 inhibitors to decrease PAR levels in PARG-null cells, with ABT-888 providing the most efficacious effect.

### Analysis of PAR levels after the short-term treatment of PARP-1 inhibitors in PARG-null cells

**Figure 2.**
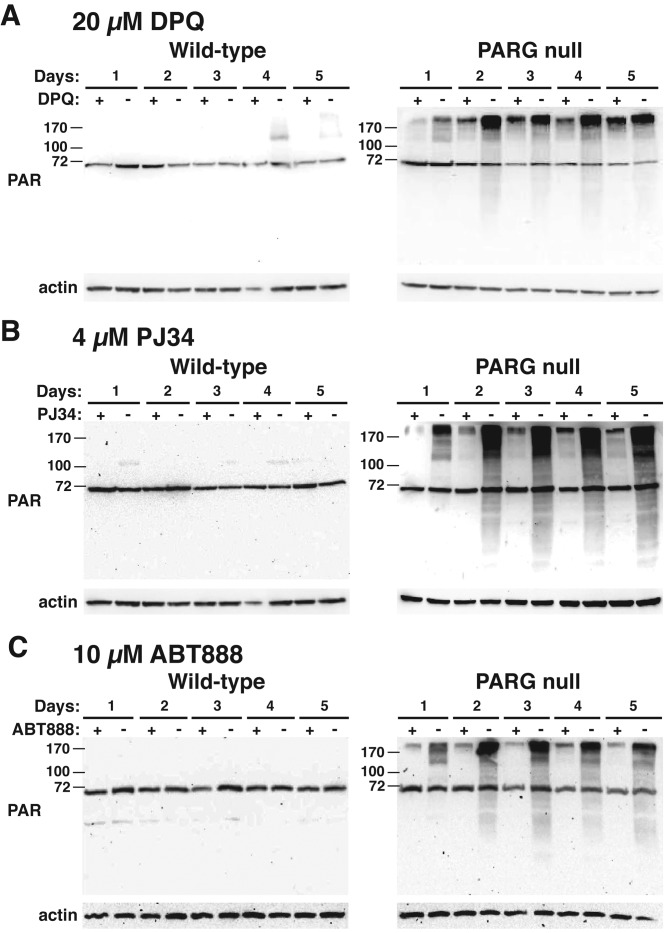
Determination of the short-term ability of PARP-1 inhibitors to prevent PAR synthesis in PARG-null cells. Wild-type and PARG-null TS cells were cultured in growth medium with or without: (A) 20 *μ*M DPQ; (B) 4 *μ*M PJ34 or (C) 10 *μ*M ABT-888 for 1–5 days. Fresh medium containing PARP-1 inhibitor was provided every 2 days. The cells were harvested each day and PAR levels were determined by immunoblotting. Loading controls for protein levels was provided by the immunodetection of β-actin.

Based on the results from Fig. 1, a dose for each PARP-1 inhibitor was selected and utilized to determine the short-term (1–5 days) effect on PAR levels. No significant accumulation of PAR was observed in wild-type cells ± treatment with PARP-1 inhibitor (Fig. 2A–C) (Note: the protein evident after immunodetection in all lanes of the wild-type and PARG-null cell PAR immunoblots is bovine serum albumin, a protein that served as a hapten for PAR in the generation of the anti-PAR antibody). However, in PARG-null cells treated with 20 *μ*M DPQ, a time-dependent increase in PAR levels was evident (Fig. 2A). Accordingly, peak levels of PAR in PARG-null cells treated with DPQ were observed at day 5. In the absence of DPQ, PAR levels were significantly higher at day 1 and peak levels were observed at day 2, which demonstrates the failure of these cells to hydrolyze PAR due to the absence of PARG. Because PAR levels in DPQ-treated PARG-null cells were lower than those observed without treatment, this indicates that DPQ inhibits the majority of PAR synthesis in PARG-null cells. Similar results were observed in PARG-null cells treated with PJ34, where PAR levels progressively increased from days 1–5 (Fig. 2B). However, in PARG-null cells treated with ABT-888, PAR levels remained suppressed from days 1–5 (Fig. 2C), which indicates the enhanced ability of ABT-888 to inhibit PAR synthesis as compared to DPQ and PJ34. Taken together, the results show the ability of DPQ, PJ34 and ABT-888 to reduce PAR levels in cells devoid of PARG. Further, the results demonstrate the enhanced ability of ABT-888 to inhibit PAR levels in PARG-null cells.

### Analysis of PAR levels and viability after the long-term inhibition of PARP-1 and the absence of PARG

**Figure 3.**
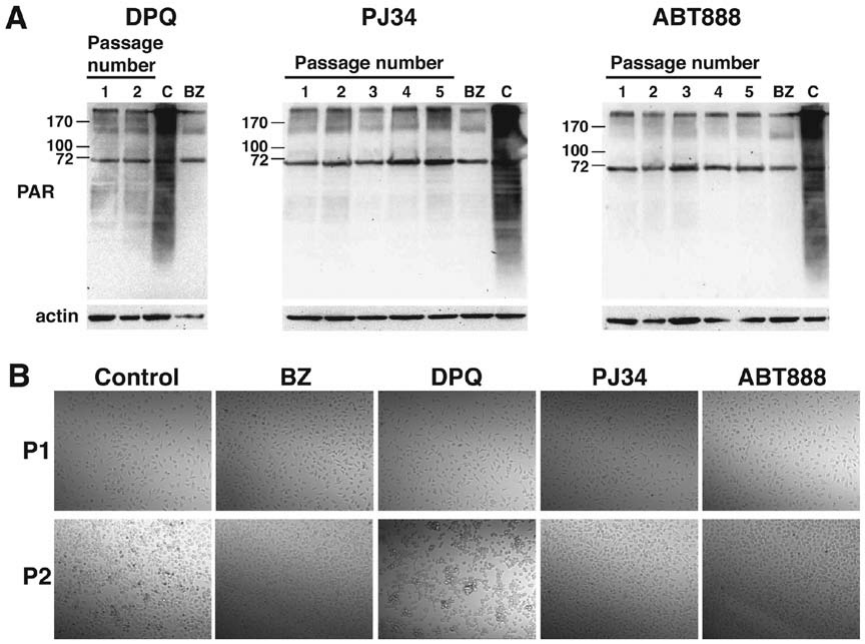
Determination of the long-term effect of PARP-1 inhibitors on PAR levels and viability in PARG-null cells. (A) PARG-null TS cells were cultured and treated with PARP inhibitors as in Fig. 2 and maintained for 5 passages. At the end of each passage, cells were collected and analyzed for PAR levels by immunoblot. Cells treated with DPQ only survived through 2 passages. Loading controls were provided by immunodetection of β-actin. Positive controls for PARP inhibition (BZ treatment) were equivalent as in Fig. 1. Untreated cells, which served as negative controls (C), were collected after passage 2. (B) At the beginning (the day after plating) of passages 1 (P1) and 2 (P2), cells were viewed by light microscopy.

To determine the long-term effects of PARP-1 inhibition in PARG-null cells, PARG-null cells were treated with PARP-1 inhibitors for up to five passages (for a total of 15 days). During treatment with DPQ, PAR levels remained significantly decreased as compared to PAR levels in PARG-null cells with no PARP-1 inhibitor (Fig. 3A). However, by the end of passage 2, the number of DPQ-treated PARG-null cells was decreased (Fig. 3A), and no cells remained viable during passage 3. In PARG-null cells treated with PJ34 or ABT-888, PAR levels were decreased at the end of each passage as compared to untreated cells (Fig. 3A). In agreement with the results in Fig. 2, treatment with ABT-888 led to the greatest reduction of PAR levels in PARG-null cells after each passage. In contrast to DPQ, both PJ34 and ABT-888 led to prolonged viability in PARG-null cells, since these cells remained viable for up to 5 passages and they exhibited no abnormalities at the conclusion of these experiments. Visual analysis by light microscopy revealed no obvious abnormalities in growth or morphology in passages 1 and 2 (Fig. 3B). These results thus indicate that the PARP-1 inhibitors, PJ34 and ABT-888, can lead to the long-term supression of PAR, as well as long-term viability in cells that are devoid of PARG catalytic activity.

### Analysis of cell death in response to chemotherapy after inhibition of PARP-1 and the silencing/absence of PARG

**Figure 4.**
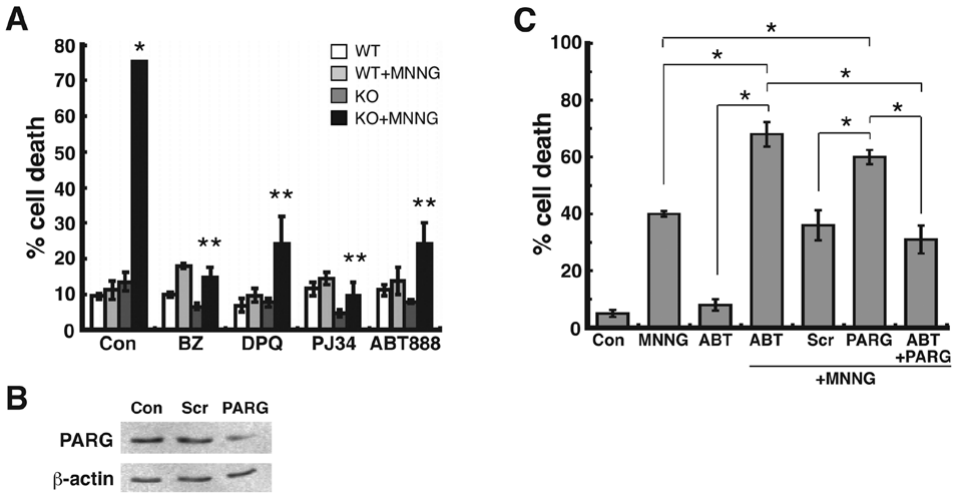
Effect of PARP-1 inhibition and knockdown/absence of PARG on cell death induced by chemotherapeutic treatment. (A) Wild-type (WT) and PARG-null (KO) cells were cultured ± PARP inhibitor as in Fig. 2 for 3 days. Cells were then treated with 50 *μ*M MNNG for 10 min. After 24 h, cell death was analyzed by FACS. ^*^P<0.05 KO vs. KO + MNNG; ^**^P<0.05 untreated KO + MNNG vs. PARP-1 inhibitor + KO + MNNG by one-way ANOVA and Student’s t-test. (B) HeLa cells were transfected with small interfering RNA oligos for PARG as described in Materials and methods. After 48 h post-transfection, immunoblotting detection of PARG in cell extractswas performed using polyclonal anti-PARG antibody (1:1,000 dilution). Controls were provided by untransfected cells (Con) and cells transfected with scrambled siRNA oligos (Scr). β-actin provided the loading controls. (C) HeLa cells were pretreated with 10 *μ*M ABT-888 and/or RNAi silencing of PARG as in (B). Cells were then treated with 0.5 mM MNNG for 30 min. After 24 h, cell death was analyzed by FACS. All error bars represent the SEM. ^*^P<0.05 by one-way ANOVA and Student’s t-test.

To determine the effect of PARP-1 inhibition and the absence of PARG on cell death induced by DNA-damaging chemotherapeutic treatment, we treated PARG-null cells with DPQ, PJ34 or ABT-888 and induced DNA damage using the experimental chemotherapeutic methylating agent, MNNG (39). The results demonstrate that the cell death due to the absence of PARG (75%) is significantly reduced by pretreatment with DPQ, PJ34 and ABT-888 (Fig. 4A). Because the dose of MNNG utilized in these experiments (50 *μ*M) was lower than that normally utilized in other cell types (300–500 *μ*M), we then determined the effect of PARG RNAi silencing and PARP-1 inhibition in HeLa cells using 0.5 mM MNNG. Immunoblot analysis of PARG levels revealed that RNAi knockdown led to approximately a 60% decrease in PARG protein levels in HeLa cells (Fig. 4B). Treatment of HeLa cells with 500 *μ*M MNNG after PARG RNAi silencing led to a 70% increase in cell death (Fig. 4C). Pretreatment with ABT-888 alone led to a similar increase in HeLa cell death after MNNG treatment. However, combination pretreatment of HeLa cells with ABT-888 and PARG RNAi silencing did not lead to an increase in cell death after MNNG treatment. Taken together, the results indicate that the inhibition of PARP-1 or PARG leads to increased HeLa cell death, but the inhibition of both enzymes does not lead to additive or synergistic increases in HeLa cell death.

## Discussion

We present evidence here that expands on our understanding of targeting PAR metabolism, via targeting PARP-1 or PARG, for enhancing the efficacy of chemotherapeutic treatments. While we show that inhibiting each significantly enhances the cytotoxicity induced in cancer cells, we report the important finding that targeting both for the improvement of chemotherapeutic efficacy may not be warranted. The possibility does exist, due to the variety of cellular effects mediated by PARP-1 and PARG, such as transcription (40), chromatin dynamics (41,42), cell death (12,43) and potential role in telomere maintenance (44), that targeting both enzymes in chemotherapy may have a synergistic effect. However, in our cancer cell model utilizing HeLa cells, the data suggests that this strategy may not be effective for treating cervical adenocarcinoma tumors (from which HeLa cells were derived). As the disruption of PARP-1 or PARG activity leads to the uncoordinated metabolism of PAR, this phenomenon may be required for enhancing the chemo-therapeutic treatment of specific types of cancers. Because we determined the effects of acute, short-term PARP-1/PARG inhibition on the chemotherapeutic treatment of HeLa cells, is also possible that different chemotherapeutic results could be seen utilizing longer exposures to the PARP-1 inhibitors and PARG RNAi.

Although we report no synergistic effect by targeting both PARP-1 and PARG in combination, this report does provide additional data that increase the feasibility of targeting PARG in cancer. We previously demonstrated that the RNAi knockdown of PARG surprisingly led to the increased survival of MCF-7 breast adenocarcinoma cells in response to chemo-therapeutic treatments (45). The results here demonstrate the opposite (and desired effect) in HeLa cells, where we show that the RNAi silencing of PARG leads to increased HeLa cell death after chemotherapy. These differences in effects between the two cancer cell lines may reflect the ability of PARG silencing to induce alternative pathways of cell death in HeLa cells, since we previously demonstrated that the absence of PARG leads to decreased caspase activation and the activation of caspase-independent cell death (43,45). The differences may also be due to differential outcomes caused by the prolonged presence of PAR synthesized by other PARPs. While several PARPs have been identified in the human genome, PARG remains as the primary enzyme that catalyzes hydrolysis of the PAR synthesized by many of these PARPs. Four PARPs have been identified to date that synthesize PAR: PARP-1, PARP-2 (5), vault PARP (34) and tankyrase (35). All are involved in essential cellular functions. It is possible that various cancer cells utilize these PARPs differently. For example, some drug-resistant breast cancer cells are known to overexpress vault PARP (vPARP) (34), the first cytoplasmic PARP discovered (PARP-1 has a nuclear localization). Thus, these cells may synthesize a significant portion of cellular PAR via vPARP. The inhibition of PARG function in these cancer cells may lead to protective effects. Therefore, although we have shown that inhibition of both PARP-1 and PARG does not increase cell death in HeLa cells, we have further shown that PARG inhibition may provide an innovative strategy to treat specific types of cancer.

Further, because PARP-1 is currently a feasible anticancer target, this study demonstrates that our PARG-null cell model can provide a new and innovative method for evaluating the drug activity of new PARP-1 inhibitors. Since these cells are devoid of PARG, they can provide a method for qualitatively and quantitatively assessing the ability of PARP inhibitors to the synthesis of PAR. For example, this report shows that ABT-888, via immunoblotting analysis of PAR levels, appears to be the most potent PARP-1 inhibitor of the three agents tested. This appears to be the case currently, since ABT-888 is the only agent of the three that has been involved in previous and ongoing clinical trials to treat breast and ovarian cancer patients.

In summary, we demonstrate that combination PARP-1 and PARG inhibition does not lead to synergistic cytotoxicity in HeLa cells. However, we do provide further insight into the feasibility of targeting PARG in cancer and the ability of utilizing our PARG-null cell model to evaluate the ability of future PARP inhibitors to inhibit PAR synthesis. Future studies involving the targeting of PARG in various types of cancer cells, as well as *in vivo* studies, will be required to further validate the chemotherapeutic value of targeting PARG.

## Abbreviations:

PAR: poly(ADP-ribose)
PARG: poly(ADP-ribose) glycohydrolase
PARP: poly(ADP-ribose) polymerase
TS cells: embryonic trophoblast stem cells
BZ: benzamide
MNNG: N-methyl-N’nitro-N-nitrosoguanidine
